# Nutritional Education in the Midwife’s Consultation Room

**DOI:** 10.3390/nu15132906

**Published:** 2023-06-27

**Authors:** M. Josefa Olloqui-Mundet, M. Mar Cavia, Sara R. Alonso-Torre, Celia Carrillo

**Affiliations:** Nutrición y Bromatología, Facultad de Ciencias, Universidad de Burgos, E-09001 Burgos, Spain; mom1001@alu.ubu.es (M.J.O.-M.); salonso@ubu.es (S.R.A.-T.)

**Keywords:** attitudes, education, knowledge, nutrition, midwives

## Abstract

Evidence of the importance of maternal nutrition during pregnancy is growing, and midwives are the healthcare professionals in charge of monitoring pregnancy. In the present review, the aim is therefore to look at the relevant contributions on midwifery and the role of midwifery in the provision of nutritional education. Searches were conducted on three databases—Medline, PubMed Central, and Web of Science—using two key search terms (“nutrition” and “midwives”) and their synonyms, for an extensive set of results. The final selection consisted of 27 original papers, most of which concerned the nutritional knowledge of midwives, their training, and their views, attitudes, and practices within the field of nutrition. According to the findings, although the midwives considered nutritional education to be part of their role and they agreed on the importance of nutrition during pregnancy, their knowledge of this topic was poor, perhaps due to inadequate nutritional education during their midwifery training. Academic programs on midwifery must therefore be reviewed, so as to increase the workload of nutrition-related subjects, in order to reinforce the corresponding knowledge bases of future midwives. In addition, based on the success of the nutritional interventions discussed in the present review, these sorts of initiatives could be of utmost importance to improve the knowledge of practicing midwives. In any case, it must be highlighted that the available evidence discussed in this review was drawn from only a few countries around the world. Further studies involving midwives of varied origins are therefore needed. Such research would be of immense assistance in the design of the corresponding nutritional interventions in the field, so as to improve the health of pregnant women.

## 1. Introduction

The nutritional status of a woman during pregnancy can have a significant impact on both the health and wellbeing of the mother and the development of the fetus, as well as the newborn baby. Both fetal growth and the first stages of human life are critical periods when the molecular, genetic, and metabolic bases that condition the subsequent development of certain diseases are established [1,2]. Evidence of the importance of maternal nutrition during pregnancy is overwhelming. Multiple studies have linked excessive weight gain during pregnancy and the consumption of certain substances (e.g., alcohol, caffeine, tobacco) with health problems for the mother and the fetus, and with complications during and after childbirth [1,2].

In the case of mothers, excessive gestational weight gain and maternal obesity will increase the risk of gestational diabetes, gestational hypertension, preeclampsia, caesarean section, and depression, and may have adverse effects on the capability of a woman to begin and to maintain breastfeeding, and to lose weight postpartum [3,4,5,6,7].

Examining child-related complications, poor nutrition during pregnancy and pre-gestational maternal obesity have been linked to higher probabilities of intrauterine growth restriction, fetal macrosomia, stillbirth, congenital abnormalities, preterm birth, and low birth weight, as well as increased risks of neonatal infections, neonatal death, and neonatal hypothermia [8]. These supposed links between chronic diseases and fetal and maternal influences have been actively debated and, indeed, confirmed in various reviews and studies [9,10,11,12,13]. In 1989, the Barker hypothesis proposed a fetal origin of many adult diseases [14].

A healthy diet during pregnancy is therefore associated with benefits for both mother and baby [15]. Gestation is an ideal time to promote healthy habits, because pregnant women feel an intrinsic motivation to do their best for their babies. In this sense, various authors have highlighted the greater willingness of pregnant women to follow healthy lifestyles, healthy nutrition habits, and to control their weight. However, low levels of nutrition knowledge among pregnant women have been reported in research into such issues as obesity, recommendations on weight gain, and weight management [16]; recommendations on fruit and vegetables and other categories of foods [17]; meal portions and serving sizes, multivitamins, and supplements; the importance of key nutrients during pregnancy; and high-risk foods in relation to mercury content and listeria [18]. Poor adherence to healthy eating recommendations during pregnancy has also been reported [19].

Receiving nutritional education during pregnancy may improve the knowledge and practices of pregnant women, which could be associated with a positive development and outcome of pregnancy [20]. In fact, the WHO recommendations are that pregnant women should be advised on the importance of healthy eating and physical activity during pregnancy, so that they remain healthy without gaining excessive weight during pregnancy [21]. However, pregnant women have not always reported receiving appropriate nutrition advice from healthcare providers [17], even though healthcare providers are aware of the importance of nutrition [22].

Midwives, especially primary-care midwives who monitor pregnancies, are ideally placed as front-line healthcare professionals to provide nutritional education to pregnant women. In this sense, early studies developed 4 decades ago had already highlighted the privileged position of midwives as a reference for pregnant women [23]. Since then, their role in nutritional education during pregnancy has been increasingly acknowledged.

Considering the above, our aim is therefore to review an extensive range of contributions on the role of midwifery in the provision of nutritional education. Our specific objectives are (i) to gather knowledge of the attitudes of midwifes towards nutritional education, (ii) to evaluate the nutrition contents of midwifery curricula, and (iii) to explore nutrition-related knowledge among midwifes.

## 2. Materials and Methods

Medline, PubMed Central, and Web of Science were the electronic databases selected to conduct the literature search. The key search terms were “nutrition” and “midwife”, limiting their presence to titles and abstracts in order to refine the search. A wide variety of synonyms or alternative search terms, in both singular and plural (e.g., nutritional, diet, dietary, dietetic, midwifing, midwifery, and midwife, among others), along with the corresponding Boolean operators were applied as strategies to broaden the findings. Additionally, the reference lists of the articles that the search returned were also inspected to identify further studies. No date limits were applied. Only topic-related restrictions were included, so that original papers dealing with the following subjects were identified: (i) attitudes of midwives towards nutrition, (ii) nutrition contents in midwifery curricula, and (iii) nutritional knowledge among midwifes.

The abovementioned search returned a total of 940 articles (477 articles were retrieved from Medline and PubMed Central, and 463 from Web of Science), which were reduced to 755 after checking for duplicates. A first reading of the titles led us to eliminate 458 articles. After reading the abstracts, a further 217 articles were eliminated from the selection. Once the full text of the remaining manuscripts had been examined, 27 original papers that matched the inclusion criteria were finally included in the literature review.

## 3. Results and Discussion

### 3.1. Overview of the Studies Included in the Review

Brief summaries of the 27 selected articles are presented in Table 1. These studies took place in various countries: Australia (nine studies), the United Kingdom (six studies), Sweden (three studies), Norway (one study), the Netherlands (three studies), New Zealand (one study), Ghana (one study), Sudan (one study), and two multi-country studies conducted in Africa. The attitudes of midwifes towards nutritional education are first discussed below. Following that discussion, we will examine the nutrition contents of midwifery curricula. Finally, the available evidence concerning nutrition knowledge among midwifes is discussed.

### 3.2. Nutrition Education Provided by Midwives

The results of some studies conducted in different countries have highlighted the agreement among midwives on the importance of nutrition during pregnancy, and that counselling on nutrition for pregnant women is an important part of the midwife’s role.

In that regard, Elias and Green [24] found that 74% of the midwives in their study in New Zealand considered nutrition during pregnancy as “very important” and 24% considered it as “important”. Furthermore, when midwives were asked about their role in the provision of nutrition counselling, 48% rated it as very significant and 46% as significant. More evidence was forthcoming from Australian midwives. Arrish et al. [25] showed that 87% of the midwives in Australia considered nutrition during pregnancy to be very important, and 76% considered that they played an important role in nutrition counselling. One year later, the same research group conducted telephone interviews with Australian midwives [26]. The results from these interviews showed that all midwives were agreed upon the high importance that nutrition has during pregnancy and its influence on women and fetal health, and on the important role they play in health promotion, providing nutrition counselling to pregnant women. They thought that their role as the front-line healthcare professionals who are closest to pregnant women and the first to enter into contact with them was very important. Willcox et al. [27] conducted face-to-face interviews with 15 midwives in Australia. Interestingly, although the results from their study also highlighted the fact that midwives felt it was their responsibility and part of their role to provide nutritional education and to promote healthy lifestyles and healthy weight gain, the authors found that the topic of gestational weight gain was a low priority for them.

Similar results have been observed in studies on European midwives. For instance, Dutch midwives confirmed that they were aware of the consequences that unhealthy eating has on both women and newborn babies. They also agreed to offer nutritional education and to educate pregnant women on controlling weight gain during pregnancy, even though they thought that it was not effective enough [28]. Midwives from the UK also recognized that giving information on healthy lifestyles was part of their role and agreed that all pregnant women needed such recommendations [29,30]. British midwives considered it necessary to offer advice to pregnant women on smoking, alcohol and drugs, domestic abuse, mental health, physical exercise, healthy diet, health foods, and weight management, although the majority were unable to rank each topic in order of importance. They stated that they offered only basic advice when feeling confident of their knowledge and, whenever possible, they referred pregnant women to specialists, although they agreed that nutrition counselling was part of their role [31].

In view of the above, midwives are aware of the importance of a healthy lifestyle during pregnancy. They considered that the midwife consultation was an ideal moment to provide nutrition-related recommendations. However, several authors reported that midwives felt a lack of confidence when discussing nutrition with pregnant women. In this regard, Australian midwives voiced a need for more education in the field of human nutrition, in order to be able to give a proper nutrition counselling [32]. Only 19% of the British midwives surveyed by Macleod et al. [33] admitted to feeling confident when providing nutrition-related recommendations—something that Soltani et al. [29] also confirmed with a different sample of British midwives.

In any case, some topics are easier to talk about than others. For instance, Lee and Garrod [31] found that British midwives felt confident when providing advice on the risks of smoking for mother and baby and the benefits of quitting smoking, although they felt unconfident when discussing illicit drug use and physical activity. In contrast, six Dutch midwives in face-to-face interviews said that they felt confident when counselling on physical activity, nutrition, and weight gain [28]. Similarly, 25–33% of midwifery students from sub-Saharan countries (i.e., Kenya, Zambia, Uganda, Tanzania, Somaliland, Malawi, and Zimbabwe) who were interviewed reported high levels of confidence when advising on nutrition and physical activity, when identifying risks for mother and fetus, and when managing complications [34]. Meanwhile, 83% of midwives from New Zealand claimed that they felt confident when discussing *Listeria monocytogenes*, although only 42% felt sufficiently confident when the topic was related to vegetarian diets [24]. Different authors found that midwives mentioned that it was especially difficult to manage obese pregnant women [35,36] because the topic is sometimes perceived as embarrassing [33], and due to a lack of skills and knowledge when talking with obese women about weight gain [37]. In this sense, midwives from Norway also considered that gestational weight gain was a difficult and uncomfortable topic to manage and was less relevant than giving advice on nutrition, physical activity, and healthy lifestyles [38]. The age and size of midwives appeared to influence their comfort when covering these issues. Interestingly, overweight or obese midwives felt hypocritical when advising on weight management [23]. There were also other circumstances of pregnant women that represented challenges for midwives: eating disorders, other cultures, language barriers, and women living in adverse socioeconomic circumstances [35,36].

Although most of the midwifes considered that the provision of nutrition-related recommendations formed part of their role, they might well adopt different approaches towards their work, possibly showing a clear connection with their aforementioned lack of confidence. Thus, several authors have attended the consulting rooms of midwives to understand how a midwifery consultation is structured and what type of nutritional support the midwives actually offer. In fact, Szwajcer et al. [39] investigated 12 booking visits lasting 35 min with primiparous Dutch women. Five minutes was used to talk about nutrition, usually starting with the topics to which pregnant women should pay great attention, including the use of folic acid and pasteurized foods, and the need to consume enough fish, vegetables, and fruits. It was not common to ask women about their nutritional doubts or questions. At a different time during the consultation, a leaflet was given to the pregnant women, usually in a pack with different materials. Women were told to check it, so as to reinforce the nutritional counselling that they had been given. The results from British midwifery consultations indicated that 99% of the midwives provided information on supplements during pregnancy, 98% offered information on healthy eating during pregnancy, and 46% also distributed leaflets. The main topics covered by midwives included foods to avoid (>90%), foods rich in iron, regular meals, and healthy eating (80% to 90%). In contrast, other topics received less attention, such as snacks, fortified foods, foods rich in calcium, and weight gain recommendations [29]. Other British midwives, even though they acknowledged their role in the provision of nutritional advice, reported only offering general information on healthy lifestyles [30]. The management of gestational weight gain in British midwifery consultations has been addressed in different studies. In this sense, pregnancy body mass index (BMI) was calculated in the booking visit, sometimes asking a woman her weight rather than weighing her. At this point, very few midwives discussed weight management, which was considered to be an emotive topic. Most midwives gave no advice on weight management to obese pregnant women, unless it was requested, because they felt uncomfortable and considered that it was a difficult issue, but they reported no problems discussing weight management with underweight pregnant women [31]. In another study focused on obese pregnant women, midwives reported that they always calculated pregnancy BMI during the first visit (87% would not do it again later in pregnancy), 73% informed each pregnant woman about her BMI category, and 42% advised them how much weight they should gain. Although 98% of them thought that weight management was very important during pregnancy for obese women, only 15% offered personalized advice depending on diet and physical activity [33]. Baron et al. [40] recorded 173 booking visits that took place in the Netherlands and found that 91.9% of the midwives offered no information on weight gain recommendations. Surprisingly, less than 20% of the Dutch midwives advised on fish in the diet and on limitations on caffeine and alcohol consumption, as well as smoking habits and physical activity recommendations. Furthermore, around 40% of the midwives made no mention of folic acid recommendations, and a very low percentage of the midwifes recognized the importance of nutrition during pregnancy. In another study, Dutch midwives reported weighing pregnant women during the booking visit (and calculating their BMI), but some of them never did so again in pregnancy, except when asked to do so. They also confirmed giving advice on healthy and safe eating, discussing nutritional topics in general, and being open to questions and doubts, although they admitted that advice on physical activity was seldom given [28].

Studies conducted in Northern European countries showed that Swedish midwives focused on preventing the associated risks of unhealthy diets in early and in mid-pregnancy (coinciding with glucose tolerance tests), using different strategies. They initially tried to understand the life and context of each pregnant woman to offer them counselling adapted to their individual needs, and then they offered support and motivations to achieve the changes. If counselling did not work properly, they then tried persuasion. If a healthy diet was still not being followed, they tried to control dietary behavior (partner commitment can help). Finally, if nothing else worked, a final option could be for the midwife to reassign responsibility to another professional and, for example, refer the pregnant women to a dietitian [35]. Swedish midwives reported establishing relationships with pregnant women and adjusting the counselling to their circumstances, avoiding making women feel uncomfortable about their weight and motivating them to follow healthier lifestyles—usually through the motivational interview method, which has had proven results when changing behaviors. The midwives reported that some pregnant women wished to achieve lifestyle changes but were unable to do so in practice. They found that emphasizing the health benefits for both mother and baby, rather than describing adverse health-related risks, was a better way to promote behavioral changes [41]. In turn, Norwegian midwives said that they gave advice on physical activity (at least once per gestation), mainly in the first consultation, and 25% also gave advice later on in pregnancy, although 7.7% never counselled on nutrition, and 6.2% never counselled on gestational weight gain [38].

Australian midwives gave no set advice on nutrition as a routine, although they always covered the topic at varying levels of intensity [26]. When counselling on nutrition, it was mostly during the booking visit (72%), while others did so on each visit (48%). They mainly gave general nutritional education, and only 2% personalized the advice [25]. However, some midwives tried to build relationships with the women whom they were counselling, helping the women to feel comfortable, which gave the midwives a good opportunity to provide in-depth nutritional information gradually [26]. Not all of the Australian midwifes discussed gestational weight management [26], and the majority delivered no leaflets on diet and/or food during pregnancy [25,32]. Interestingly, the Australian midwives offered little more than basic and general nutrition recommendations, because they considered that dietitians were the professionals who should counsel in case of complex or specific issues [26]. Bondarianzadeh et al. [32] interviewed 10 Australian midwives on *Listeria* risk recommendations. They found that advice on *Listeria* mainly occurred during the first visit while explaining the importance of a healthy diet during pregnancy. Midwives usually informed about the foods that should be avoided to prevent contamination with the bacteria. However, advice on food handling was less common. Surprisingly, 60% of the midwives considered that giving this kind of recommendation was not their responsibility, and most of the midwives who were interviewed thought that pregnancy was already too late to inform on listeria-related risks. Interestingly, midwives confirmed that they only gave basic information on listeria, with no detailed explanations of the risks or consequences, in order to avoid increasing the stress of pregnant women. Australian midwives considered that a pregnant woman should assume responsibility for gaining additional information, if needed, on the topic of listeria.

Results from a study involving 56 midwives working in a Sudanese hospital showed that 100% of the midwives weighed the pregnant women on each visit, and 98% took their blood pressure. Most midwives were well informed and gave good quality advice, covering several important topics during pregnancy for mother and baby: 100% gave advice on tetanus toxoid vaccination and breastfeeding, 98% on a balanced diet and baby vaccines, 96% on personal hygiene, and 91% on family planning. However, many midwives had difficulty recognizing some signs of risk during pregnancy, which if detected early can help to avoid or prevent many problems. Fewer than half of all the midwives identified rising fever (30%), headaches (42%), and abdominal pain (42%) as signs of risk. Better known topics were vaginal bleeding (80%), swelling of the face, hands, and legs (80%), prolonged labor (71%), fits (69%), and blurred vision (57%) [42].

According to midwives, some of the barriers that make the provision of adequate recommendations on nutrition during a consultation difficult are related to pregnant women, including a lack of interest from pregnant women [36], the attitudes of pregnant women, their lack of motivation to change, and previous knowledge [26]. Some midwives also considered that the first visit was already too late in pregnancy for nutritional education [39]. However, the most commonly highlighted barriers among midwives included a “lack of time” [25,26,27,29,30,31,33,36] and a “lack of knowledge” [25,26,33,36]. Other barriers that were mentioned were somehow related to the latter, such as a “lack of clear guidance for professionals to give advice” [29] or a “lack of resources” [26,27].

In view of the above, nutritional background and knowledge is a key point for guaranteeing the success of a midwifery consultation. In this sense, midwifery training should include training in the field of nutrition and dietetics. Whether this training is broad enough is discussed in the following section.

### 3.3. Nutrition Contents in Midwifery Training

Some of the available evidence on nutrition contents in midwifery curricula comes from studies that have directly investigated the structure of the academic programs in midwifery training. In this regard, Sodjinou et al. [43] reviewed 30 midwifery training programs in a multi-country study conducted in West Africa (Burkina Faso, Côte d’Ivoire, Guinea, Mali, Mauritania, Niger, and Senegal). The authors found that nutritional instruction in midwifery schools was 48 h on average and took place during the two-year duration of the first cycle (the second cycle consisted of clinical practice). Mostly provided within the framework of a specific nutrition course in midwifery (87% of the programs examined), it mainly contained basic nutrition (77% of the programs examined), with a short focus on public health nutrition (less than 6 h). The didactic teaching method lacked a practical approach, and the nutritional instruction in midwifery training was rated as insufficient by 55% of the respondents. In another study conducted in Australia [44], 23 midwifery course coordinators were surveyed through an online questionnaire, 7 of whom were then interviewed by telephone. Overall, the coordinators considered that the field of nutrition within the midwifery curriculum was essential, insofar as they were all agreed on the importance of providing nutritional education for a midwife. The 23 programs under evaluation included nutrition contents, although the load varied between the different programs: while 2 of them had a designated unit, 11 only devoted 5 to 10 h to nutrition issues. Various topics were covered. On the one hand, topics related to nutrition during pregnancy (i.e., alcohol and pregnancy; management of nausea and vomiting; the role of folate, iodine, and calcium during pregnancy; the healthy ranges of weight gain required for pregnant women; nutrition for breastfeeding; nutritional management of gestational diabetes; food safety and handling during pregnancy; nutritional assessment (i.e., review the diet according to the nutritional requirements of pregnancy); nutrition in teenage pregnancy). On the other hand, topics of general nutrition (i.e., general nutrition for special groups—vegetarians, vegans, and different cultural groups; general information on nutrients—the role of nutrients, vitamins and minerals in the human body; general food safety; general nutrition—for instance, prevention of chronic illnesses such as cancer and heart disease). However, despite the wide variety of contents on offer, the nutritional education was mainly focused on typical problems during pregnancy and how to solve them (e.g., preventing constipation), rather than on the provision of healthy habits. In addition, a lack of practical training to improve skills and advice was detected. Concerning the experts who were in charge of teaching these nutritional contents, it is important to highlight the rare involvement of dietitians and other experts within the field of human nutrition in these courses, which were mainly taught by midwives. This all suggests that nutritional education in midwifery programs must be reviewed, and that minimum requirements should be included to improve the effectiveness of midwives within this area, simply because nutrition is essential for maternal and fetal health—an update that might require collaboration with nutrition experts.

In addition to the aforementioned studies, some other evidence in this respect comes from the self-perceptions of midwives when asked about their nutritional background. In this regard, Mulliner et al. [45] interviewed 77 British midwifes and found that 86% had received nutritional education courses during their formal midwifery education, mostly taught by midwifes (95%). However, 66% of the British midwifes had received fewer than two sessions throughout their complete training. The results of another study among British midwifes [29] showed that only 38% of the midwives had learned about supplements during their formal education. Interestingly, most of the British midwifes who were interviewed considered that they needed more training in nutrition during their formal education and claimed that there was a need for continuous education after their academic training [29,31]. British midwives involved in the study of Lee and Garrod [31] reported having no direct training on healthy eating habits. They used their own knowledge and experience and agreed that they needed further training in all areas, especially on weight, diet, and physical activity. They acknowledged that time and costs were obstacles to accessing courses, unless some were directly available.

In another study developed in New Zealand that involved 370 midwives [24], the authors found that only 37% of the sample interviewed had received formal nutritional education during their midwifery education. In addition, focusing on topics such as weight management, nutrition assessment, and nutrition for vulnerable groups was perceived as insufficient. Similarly, results from other studies performed with Australian and Swedish midwives [27,32,36] suggested a lack of knowledge and skills to advise on nutrition, due to insufficient training in nutrition during their formal education. All of the midwives who were interviewed agreed that they needed more in-depth and ongoing training and support to improve their skills and knowledge.

Considering the above, although midwifery training differs between countries, there is generally insufficient training in the field of nutrition during midwives’ academic training. As a consequence, if midwifes are to develop their professional activities in an adequate manner, they have to look for reliable nutritional information themselves. In this respect, the main sources used by midwifes to check nutritional issues include nutritional organizations (86%), midwifery journals (64%), other professionals (53%), official institutions, and medical authorities [24,36,46]. Education in the field of nutrition throughout their professional career occurred mainly through personal initiatives, by enrolling on external courses, exploring the Internet, or holding discussions with colleagues. Whether this continuous training is sufficient to pass on adequate nutritional knowledge among midwifes will be discussed in the next section.

### 3.4. Nutritional Knowledge of Midwives

To date, several authors have evaluated the nutritional knowledge of midwives. In this respect, Mulliner et al. [45] used a postal survey (blind validation of the knowledge section), which was followed up with an interview, to explore the nutritional knowledge of 58 British midwives. The authors found that 46% of the midwives achieved a poor score in nutritional knowledge. This percentage had no relation with other issues, such as the type of midwifery program, the range of professional experience, or how much time they devoted to treating nutritional topics with pregnant women. Furthermore, midwives said that they felt unprepared to offer dietary advice to women from ethnic minority groups (36% of the responders), to vegetarian women (66%), and to women with diabetes or gestational diabetes (41%), and that they required further nutrition-related education during and after their midwifery education. In a more recent study, McCann et al. [30] also interviewed 17 British midwives, in order to assess their nutritional knowledge. Most of them did not know the requirements of the different nutrients during pregnancy, apart from folate and iron. Surprisingly, although they were aware of the risks to mother and fetus posed by a high BMI prior to gestation, they could not properly identify the ranges of healthy weight gains according to previous BMI. Poor knowledge of nutrition has been observed in other study from the United Kingdom [31].

Merkx et al. [28] interviewed six Dutch midwives, five of whom were unaware of the Institute of Medicine (IOM) guidelines, the risks of insufficient or excessive weight gains, and that weight gain should depend on the initial weight. In addition, their knowledge of healthy eating and physical activity was also superficial.

Outside Europe, evidence concerning the nutritional knowledge of midwives has come from New Zealand and Australia. Elias and Green [24] conducted a (pilot-tested) postal survey, which involved 370 midwives in New Zealand, in order to evaluate different issues related to the nutritional education in midwifery consultations (response rate: 27.6%). Nine of the questions included in the questionnaire were intended to assess the nutritional knowledge of midwives. The level of correct answers depended on the question. For instance, the majority of midwives knew of issues such as “iron sources for vegetarians”, “Vitamin C enhances iron absorption” (96.8%),“iron absorption is inhibited by tea during meals” (93.8%), “listeria risk foods (soft cheese and ready to eat salads widely identified as high risk of listeria” (90.3% and 95.9%, respectively), “energy requirements higher in lactation than pregnancy” (78.9%), “spirulina as invalid source for B12 vitamin” (54.9%), and “vegetables and fruits as poor sources of zinc” (54.9%). In contrast, a low percentage of midwives were aware of “weight gain recommendations” (41%) and that “breast milk is a poor source of vitamin D” (32.2%). Arrish et al. [25] carried out a web-based survey in Australia completed by 329 midwives. The questionnaire included 12 questions to measure knowledge of nutrition during pregnancy among midwifes: 8 single-answer questions (energy requirements for pregnant women; whether those requirements differed between the three trimesters; weight gain for women with a normal pre-pregnancy BMI; which vitamin(s) must be supplemented if vegetarian; when and what amount of folic acid supplements must be taken; recommended portions of dairy foods to achieve the calcium requirements during pregnancy; and daily iodine needs for pregnant women) and 4 multiple-answer questions (foods to avoid for listeria risk; sources of iron; how to decrease vomiting and nausea; and foods to improve constipation), each with three correct answers. Concerning the single-answer questions, the best result was observed for the question on when pregnant women should take folic acid (94% right), followed by the amount of daily folate needed (67% right), whether energy requirements differed between the three trimesters (64% right), and which vitamin(s) must be supplemented for vegetarians (62% right). In contrast, the questions with the most wrong answers were related to daily iodine needs for pregnant women (80% wrong) and recommended weight gain for women with a normal pre-pregnancy BMI and recommended portions of dairy foods for acceptable calcium intake during pregnancy (in both cases, 73% wrong). In the multiple-choice questions, 78% of the responders identified the three foods listed to lessen the risk of listeria infection (soft cheeses, pre-prepared salads, cold meats); 83% identified one or more of them independently; only 9% identified all four sources of iron on the list (over 93% each selected red meat and green leafy vegetables); 64% ticked all three correct items listed to decrease vomiting and nausea (the best-known item was eat something solid before getting out of bed); and 95% chose all of the correct foods listed to improve constipation. The Australian midwives had an average score of 14.96 out of a maximum 20 points (a minimum score of 7 and a maximum of 18). The results indicated that midwives working in the private sector had higher scores than midwives within the public sector (14.75 and 13.54, respectively); likewise, midwives working in regional rather than in rural hospitals (13.96 and 12.92, respectively), midwives who had received nutritional education during their midwifery education rather than those who had received none (13.96 and 13.30, respectively), and midwives with higher levels of confidence at providing nutrition education than midwives with moderate or low confidence levels (14.48, 13.70 and 13.15, respectively) all had better scores. A poor knowledge of nutrition was observed in other studies on Australian midwives [37].

Information from Africa on midwifery practice is largely available through studies centered on midwifery students. For instance, Nsiah-Asamoah and Ampofo [47] administered a survey on nutritional knowledge to midwifery students from the African continent. They investigated the nutritional knowledge of 562 students (last year of training) from Ghana, using a questionnaire with 20 multiple-choice questions on nutrition during pregnancy. The authors found that less than 50% of the students correctly answered 11 of the 20 questions included in the test—some of them as important as the strong association between folates and the prevention of neural tube defects (49.8% right answers); the 400 μg daily folic acid recommendations during pregnancy (46.6% right answers); that bananas are not a good source of iron (39.5%); that milk is not a good source of folic acid (44.5%); that the screening for gestational diabetes should take place around 24/28 weeks (39.5% right answers); or that vitamin D works with calcium to develop the bones of a baby (38.8% right answers). Moreover, less than 20% of the students correctly answered three other questions on whether folate deficiency during pregnancy can produce megaloblastic anemia (17.4% right); what the weight gain recommendations for normal BMI women should be (12.5%); and whether the daily iron requirements during pregnancy should be 27 mg (9.6%). The last question required particular attention, because the WHO reported in 2017 that over 40% of pregnant women worldwide suffered from anemia, and that at least half of all cases were due to iron deficiency. The best percentages were found for knowledge on alcohol risk (86.8% right), recommendations to improve constipation (85.4%), and when to start supplementing folic acid (74.7%). On average, the authors rated the nutritional knowledge of the students as 9.8 out of 20, which means that 49% of the students showed poor nutritional knowledge, which is consistent with another study from the African continent carried out in Sudan, where researchers found that midwives had poor knowledge of nutrition, based on poor skills at identifying food sources of iron or calcium (specific data not provided) [42]. These results may be attributed to the lack of emphasis or priority given to nutritional education during midwifery training [43,44].

In view of the above, it has been suggested in many studies to date that midwives may generally have significant knowledge gaps within the field of food science and nutrition. Taking into account that adequate nutritional knowledge may guarantee the success of nutritional education for pregnant women, several authors have investigated the efficacy of nutritional training on the nutritional knowledge of midwives and their related confidence levels. In this regard, Basu et al. [48] gave a compact training (3.5 h long) on nutrition, physical activity, and weight management during pregnancy, to increase the knowledge and confidence of British midwives in this field. Thirty-two midwives attended the training and completed the questionnaires. Midwives were asked to rate (from 1 (low) to 10 (high)) their knowledge in response to different statements (knowledge of the range of risks related to obesity; pregnancy-specific foods and nutrition messages based on the Eatwell Plate; recommended vitamins during pregnancy; benefits of physical activity; weight gain recommendations; how to talk about behavioral changes during pregnancy) before the training. Following the training, they had to indicate the improvements on a Likert scale (“much better/some better better/little better/stayed the same”). In all, 59% of the midwives reported having limited knowledge of nutrition, physical activity, and gestational weight management, but the scores varied significantly between the different statements that were shown. Interestingly, all of the midwives showed higher knowledge levels after receiving the training. For instance, 97% of the midwives improved their knowledge related to the field of “pregnancy specific foods and nutrition messages”, which in the pre-training had an average rating of 6 out of 10; 91% improved in relation to both “recommended weight gain during pregnancy” and “tips to start conversations with pregnant women to promote dietary change and physical activity”; 89% of the midwives also confirmed that their knowledge of a range of obesity risks had increased.

In another study carried out in Australia, de Jersey et al. [49] offered a 40 min training on healthy diets, including topics such as weight gain recommendations, physical activity, healthy eating, and healthy lifestyles. Questionnaires were completed by 153 (pre-training questionnaire) and 112 (post-training questionnaire) midwives. The authors aimed first to evaluate the perceived knowledge of midwives before the training, using seven items (rated from 1 = not expert at all to 10 = highly expert), including risks associated with high BMI for the mother (average rating 7.1) and for the baby (average rating 6.7); recommended weight gains depending on previous BMI (average rating 6.6); Australian guidelines of nutritional recommendations for pregnant women (average rating 6.0); recommendations for physical activity (average rating 6.4); and weighing pregnant women (average rating 7.1). An improvement in perceived knowledge on all seven items after training—from 71% to 97%—was observed among the midwives. Along the same lines, the perceived confidence in all of the items increased from 83 to 90%. The participants really appreciated the training that they had received; 95% would recommend it, mainly for the communication skills provided, and 85% found that the information they had received was relevant for counselling pregnant women on nutrition. In addition to the perceived nutritional knowledge, the authors also evaluated the knowledge of midwifes using an objective scale of eight items on three topics (gestational weight gain, nutrition, and physical activity). The results showed poor knowledge in the pre-training: knowledge on gestational weight gain, nutrition, and physical activity scored 11 out of a maximum of 17. The post-training scores were 15 out of a maximum of 17. Improvement was observed in gestational weight gain and nutrition, whereas knowledge relating to physical activity remained the same. Therefore, the overall objective knowledge increased after the training (from 11 to 15 out of 17). These results indicate that increasing nutritional education for midwives can greatly increase their knowledge and confidence.

Once again in Australia, Othman et al. [50] recently measured the knowledge of 44 midwives before and after two educational interventions (online webinar or workshop). They used a survey with 12 multiple-choice questions based on validated questions from other questionnaires. The knowledge of the midwives was assessed at three points: before the training (pre), immediately after the training (immediate), and at 6–8 weeks post-training. In the pre-training test, over half of the midwives only answered four questions correctly: “low glycemic index sources” (88.6%), “protein sources” (65.9%), “most important vitamins for vegetarians” (60.5%), and “sources of fiber” (52.3%). In contrast, a high percentage of incorrect answers was observed for issues such as “energy requirement” (95.5%), “vitamin D requirement” (88.6%), “sources of omega-3” (75%), “iodine requirements” (72.7%), “folic acid requirement” (61.4%), “daily water intake” (59.1%), “calcium for vegans” (54.5%), and “recommended weight gain” (52.3%). Whereas approximately half of the midwives answered just 4 out of 12 questions correctly before the training, great improvements were noted immediately after the training, when over half of midwives correctly answered 10 out of 12 questions. In the 6–8-week follow-up, a drop in the knowledge was observed, with 8 out of 12 right answers. Some topics, including “energy requirement” and “fiber sources”, showed low rates of improvement, while others, such as “vitamin D and omega-3 requirements”, improved immediately after training, although the improvements were not maintained at the follow-up. Therefore, education on healthy eating produced a statistically significant improvement in midwifery knowledge (from 4.93 to 7.55 out of 12 immediately after the training, and 7.32 out of 12 in the follow-up test 6–8 weeks later). The results also highlighted better scores when midwifes followed the training through the workshop than through the online webinar.

In view of the above, it can be said that few studies have been performed to evaluate the nutritional knowledge of midwives, but they all concur insofar as that knowledge is mostly poor and insufficient. For instance, issues related to weight gain recommendations are repetitively highlighted among the available studies as topics that are especially unknown among midwives. In contrast, tips to avoid constipation or listeria-related foods are better controlled. According to the success reported in different studies, nutritional interventions focused on active midwives might improve both their knowledge and confidence, and they are therefore suggested as an alternative to confront the problem detected in the short term.

## 4. Conclusions

Despite the growing evidence indicating that dietary advice might guarantee adequate gestational weight gain and may reduce the incidence of some poor pregnancy outcomes, the provision of antenatal nutrition advice during pregnancy is not a common practice. According to the literature reviewed in the present work, midwives were found to have positive attitudes towards nutrition during pregnancy, and they acknowledged their role as educators in nutrition. However, they reported inadequate educational background in the field of food science and nutrition, along with a lack of confidence when providing nutritional support to pregnant women, in all likelihood due to a lack of knowledge in the field.

In view of the above, it would be important to review the academic programs on midwifery and to increase the workload of nutrition-related subjects, in order to reinforce the corresponding knowledge bases of future midwives. In addition, in view of the successful nutritional interventions discussed in the present review, these kinds of initiatives could be of the utmost importance to improve knowledge among existing midwives.

Nevertheless, it is important to highlight that, as far as the authors are concerned, there is limited research in this field. The midwife, who is ideally placed to provide nutritional advice to pregnant women, has been the focus of scarce research so far. For instance, studies in Asia or America are lacking. Moreover, the knowledge concerning European midwives is limited to few countries, such as the UK, Sweden, the Netherlands, and Norway. Other limitations of the available evidence come from the small sample size selected in some of the studies and the use of non-standardized or validated tools, which complicate any interpretation and comparison of the results. Further studies involving midwives from different origins—aimed at exploring their training in nutrition, the importance they give to nutrition during pregnancy, what they think of their role as providers of nutritional education, or how comfortable they feel when addressing these nutritional issues—are therefore needed. Such research might be of help in the design of the corresponding nutritional interventions in the field, in order to improve the health of pregnant women.

**Table 1:** Key characteristics of the studies included in the review.

Country	Aim of Study	Method and Sample Size	Sample Size	Response Rate	Reference
Australia	To explore the education of midwives on listeria-related risks during pregnancy.	Exploratory design with a qualitative framework In-depth semi-structured interviews	10	NI/NA	Bondarianzadeh et al. [32]
Australia	To explore the experiences and the concerns of midwives when attending to obese women.	Descriptive qualitative. Focus groups and face-to-face recorded interviews	34 midwives (plus 3 other professionals)	NI/NA	Schmied et al. [37]
Australia	To explore how midwives manage and promote healthy weight gain during pregnancy.	Qualitative descriptive. Face-to-face interviews using an interview guide	14	48.3%	Willcox et al. [27]
Australia	To explore nutritional education in Australian midwifery programs.	Mixed methods. Quantitative and qualitative. Online survey and telephone interviews. Reviewed and piloted.	23 course coordinators	56.1%	Arrish et al. [44]
Australia	To investigate knowledge, attitudes, and confidence among Australian midwives advising on nutrition during pregnancy.	Web-based survey. Reviewed and piloted.	329	6.9%	Arrish et al. [25]
Australia	To assess the role of Australian midwives and their perceptions when providing nutritional advice, as well as any barriers to and facilitators of that role.	Qualitative, descriptive. Semi-structured telephone interviews.	16 (subsample of (25))	NI/NA	Arrish et al. [26]
Australia	To assess the nutritional education received by Australian midwives before and after registration.	Quantitative and qualitative data. Online survey.	329	6.9%	Arrish et al. [46]
Australia	To evaluate the knowledge of midwives and improvements in their confidence after a healthy weight gain and management training session.	Self-administrated questionnaire pre- and post-training session.	154% pre 114% post	74% pre. 55% post	de Jersey et al. [49]
Australia	To assess the knowledge and confidence of midwives when discussing healthy eating during pregnancy.	Pre- and post-intervention and follow-up at 6–8 weeks. Semi-structured questionnaires. Piloted for validity and reliability.	Pre- and post-intervention, and follow-up questionnaires administered to 44, 29, and 19 midwives, respectively	NI/NA	Othman et al. [50]
Ghana	To explore the nutritional knowledge of midwifery students at six diploma-awarding midwifery institutions.	Descriptive cross-sectional study. Pre-tested self-administered questionnaire	562	98.4%	Christiana and Evelyn [47]
Multi-country/West Africa	To explore nutritional training at midwifery schools in West Africa (Burkina Faso, Côte d’Ivoire, Guinea, Mali, Mauritania, Niger, and Senegal).	Semi-structured questionnaire.	30 midwifery training programs reviewed	NI/NA	Sodjinou et al. [43]
Multi-country/Africa	To explore the confidence of midwifery students when practicing different antenatal skills.	Self-administered questionnaire.	1407 midwifery students	NI/NA	Hildingsson et al. [34]
Netherlands	To assess the range of information on healthy behavior that midwives share with pregnant women during the prenatal booking visit.	Quantitative. Video-recorded prenatal visits with midwives.	173 recorded booking visits	NI/NA	Baron et al. [40]
Netherlands	To explore verbal and written nutritional communication among Dutch midwives.	Qualitative. Audio-recorded primiparous booking visits.	12	NI/NA	Szwajcer et al. [39]
Netherlands	To explore how primary-care Dutch midwives promote healthy gestational weight gain (GWG), and their related attitudes, confidence, and knowledge.	Qualitative face-to-face interviews with primary-care midwives.	6	NI/NA	Merkx et al. [28]
New Zealand	To explore the nutritional knowledge of New Zealand midwives and the importance they attach to nutrition in pregnancy.	Postal survey pilot tested with several midwives.	370	27.6%	Elias and Green [24]
Norway	To ascertain opinions and practices of midwives to promote healthy GWG, regular physical activity (PA), and nutrition.	Electronic survey.	65	60.7%	Haakstad et al. [38]
Sudan	To explore midwives’ knowledge of antenatal care.	Standardize pre-tested questionnaire (closed and open questions).	56	NI/NA	Elmoneim et al. [42]
Sweden	To explore dietary counselling strategies among midwives as well as when and what kind of dietary advice is given to pregnant women.	Qualitative. Telephone interviews.	17	77.3%	Wennberg et al. [35]
Sweden	To report perceived roles of midwives and their influence on the dietary advice given to pregnant women.	Qualitative. Face-to-face interviews	21	NI/NA	Wennberg et al. [36]
Sweden	To find out how midwives counsel high pregnancy body mass index (BMI) to achieve a healthy lifestyle and to minimize weight gain.	Qualitative. Semi-structured interviews recorded (50% face-to-face, 50% telephone).	16	NI/NA	Olander et al. [41]
UK	To explore education, knowledge, and attitudes of English midwives on nutrition in pregnancy.	Quantitative and qualitative. Postal questionnaire and semi-structured interview.	58	78%	Mulliner et al. [45]
UK	To explore opinions and practices on health in pregnancy among midwives.	Interviews.	13	NI/NA	Lee and Garrod [31]
UK	To explore practices, knowledge, and views on weight management for obese pregnant women.	Semi-structured web-based piloted questionnaire.	78	32%	Macleod et al. [33]
UK	To explore information and advice on diet and supplements before, during, and after pregnancy.	Qualitative. Online questionnaire. Previously piloted.	146 antenatal healthcare providers, mainly midwives	NI/NA	Soltani et al. [29]
UK	To research perceptions, knowledge, and experiences of midwives when providing pregnant healthy eating and weight management advice.	Qualitative. Semi-structured interviews with community and hospital midwives.	17 (6 community/ 11 hospital midwives)	NI/NA	McCann et al. [30]
UK	To give training on nutrition, PA, and weight management to improve midwives’ knowledge and confidence	Pre- and post-training questionnaires on self-reported knowledge, and confidence levels.	32	74%	Basu et al. [48]

Sample size refers to the number of individuals participating in the study. NI and NA refer to “Not Indicated” in the study and to “Not Applicable” to the design of the study, respectively.

## Data Availability

No new data were created in this study.
